# 1371. Results From the COVID-19 Vaccines Discrete Choice Experiment Pre-Test Qualitative Interviews in Canada, Germany, the UK, and US General Population

**DOI:** 10.1093/ofid/ofad500.1208

**Published:** 2023-11-27

**Authors:** Sumitra Sri Bhashyam, L G Shane, Hannah B Lewis, Marie de la Cruz, Jayne Galinsky, Keeva Demchuk, Nancy M Waite, Jeffrey V Lazarus, David M Salisbury

**Affiliations:** ICON, Reading, England, United Kingdom; Novavax Inc., NYC, New York; ICON, Reading, England, United Kingdom; ICON plc, ALEXANDRIA, Virginia; ICON, Reading, England, United Kingdom; ICON, Reading, England, United Kingdom; University of Waterloo, Mississauga, Ontario, Canada; ISGlobal, Hospital Clínic, University of Barcelona, Barcelona, Spain; Royal Institute of International Affairs, Chatham House, London, London, England, United Kingdom

## Abstract

**Background:**

COVID-19 vaccine preferences can influence vaccine coverage. Discrete choice experiments (DCE) can be used to elicit people’s trade-offs. DCEs require evidence-based attribute selection and validation of understanding with lay audiences. To inform a future DCE, a pre-test was conducted to examine the survey and refine six attributes selected from a targeted literature review and expert interviews.

**Methods:**

Interviews were conducted in March 2023 in Canada, Germany, the UK, and US. Self-reported anti-vaccinationists were excluded. Eligible individuals were interviewed during the completion of a survey that included 11 choice tasks and supplementary questions. The “think aloud” method was used to evaluate participants’ understanding of the survey and if they were making trade-offs as hypothesized. Four country-level experts validated the survey modifications based on the results.

**Results:**

Six phone interviews were completed in each country (N=24). Mean age was 43.7; 50% were women; 50% reported receiving the full COVID-19 vaccine series; 45.8% received the initial series but were unsure about additional doses; 1 was unvaccinated (4.2%).

Participants’ top four priorities were vaccine protection against COVID-19, serious side-effects, protection against severe COVID-19, and common side-effects, followed by vaccine type and timing of COVID-19/influenza vaccines (Fig 1).

More than half of the participants would consider co-administration of COVID-19 and influenza vaccines, either as two separate injections (58.3%) or as a single, combined injection (62.5%) (Fig 2a). Most individuals (54.2%) preferred an annual COVID-19 vaccine; over every 6 months (4.2%), and 20.8% were indifferent (Fig 2b).

When deciding to get vaccinated, most considered the following to be important: how long a vaccine was examined in humans (65.2%), how long a vaccine was used in a vaccination program (62.5%); 50% considered vaccine type (mRNA or protein subunit) important (Fig 3).

**Figure:**
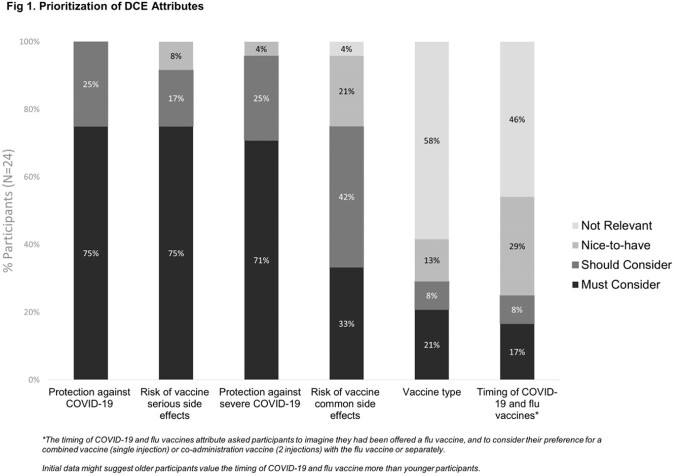

**Figure:**
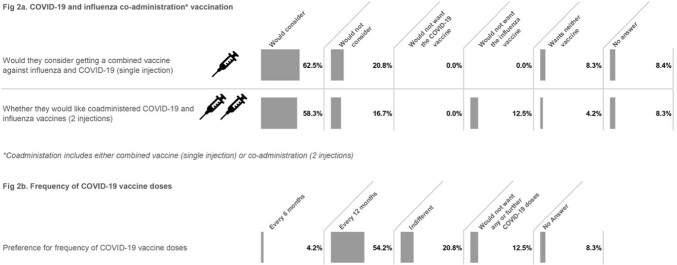

**Figure:**
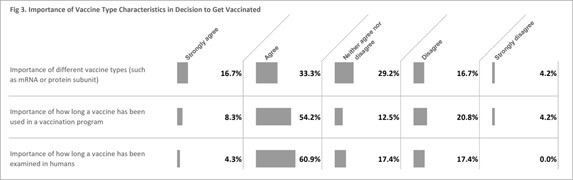

**Conclusion:**

This study validated the importance of key vaccine attributes driving people’s choices and feedback was used to improve the clarity of attribute descriptions. A future DCE will be fielded to increase the understanding of COVID-19 vaccine preference and hesitancy.

**Disclosures:**

**Sumitra Sri Bhashyam, MSc, PhD**, Novavax Inc: Grant/Research Support **L.G Shane, Pharm.D., RPH., BScPharm.**, Novavax Inc: Employee of Novavax Inc|Novavax Inc: Stocks/Bonds **Hannah B. Lewis, MSc, PhD**, Novavax Inc: Grant/Research Support **Marie de la Cruz, MS**, Novavax Inc: Grant/Research Support **Jayne Galinsky, PhD**, Novavax Inc: Grant/Research Support **Keeva Demchuk, n/a**, Novavax Inc: Grant/Research Support **Nancy M. Waite, Waite PharmD FCCP**, GSK: Advisor/Consultant|Novavax Inc: Honoraria|Pfizer: Advisor/Consultant|Sanofi: Advisor/Consultant|Sanofi: Grant/Research Support **Jeffrey V. Lazarus, PhD, MIH, MA**, AbbVie: Advisor/Consultant|AbbVie: Conference travel|Gilead Sciences: Advisor/Consultant|Gilead Sciences: Grant/Research Support|Gilead Sciences: Honoraria|Moderna: Honoraria|Novavax Inc: Advisor/Consultant|Novavax Inc: Honoraria|Novo Nordisk: Honoraria|Roche Diagnostics: Grant/Research Support **David M. Salisbury, CB FMedSci FRCP FRCPCH FFPH**, Clover Pharmaceuticals: Advisor/Consultant|GSK: Advisor/Consultant|Moderna: Advisor/Consultant|Novavax Inc: Honoraria|Sanofi: Advisor/Consultant

